# Persistent Urogenital Schistosomiasis and Its Associated Morbidity in Endemic Communities within Southern Ghana: Suspected Praziquantel Resistance or Reinfection?

**DOI:** 10.3390/medsci8010010

**Published:** 2020-02-10

**Authors:** Patience B. Tetteh-Quarcoo, Peter O. Forson, Seth K. Amponsah, John Ahenkorah, Japheth A. Opintan, Janet E. Y. Ocloo, Esther N. Okine, Robert Aryee, Emmanuel Afutu, Abraham K. Anang, Patrick F. Ayeh-Kumi

**Affiliations:** 1 Department of Medical Microbiology, School of Biomedical and Allied Health Sciences, College of Health Sciences, University of Ghana, Accra 233, Ghana; petsonbiomed2015@yahoo.com (P.O.F.); jaopintan@ug.edu.gh (J.A.O.); bobby200055@gmail.com (R.A.); eafutu@ug.edu.gh (E.A.); pfayeh-kumi@ug.edu.gh (P.F.A.-K.); 2 Department of Pharmacology and Toxicology, School of Pharmacy, College of Health Sciences, University of Ghana, Accra 233, Ghana; sethicom@yahoo.com; 3 Department of Anatomy, School of Biomedical and Allied Health Sciences, College of Health Sciences, University of Ghana, Accra 233, Ghana; daajohnny@yahoo.com; 4 Department of Pathology, Korle-Bu Teaching Hospital, Korle-Bu, Accra 233, Ghana; janeteocloo@gmail.com; 5 Central Laboratory Services, Korle-Bu Teaching Hospital, Korle-Bu, Accra 233, Ghana; gyister7@yahoo.co.uk; 6 Department of Physiology, School of Biomedical and Allied Health Sciences, College of Health Sciences, University of Ghana, Accra 233, Ghana; 7 Department of Parasitology, Noguchi Memorial Institute for Medical Research, College of Health Sciences, University of Ghana, Accra 233, Ghana; kbosompem@ug.edu.gh

**Keywords:** *Schistosoma haematobium*, praziquantel, reinfection, resistance, urine

## Abstract

Background: schistosomiasis is a neglected tropical disease caused by helminths of the genus *Schistosoma*. The disease has a worldwide distribution, with more cases occurring in Africa. Urogenital schistosomiasis caused by *S. haematobium* with its associated morbidity is prevalent in many areas of Ghana. Praziquantel is still the recommended drug of choice for schistosomiasis treatment, although a number of studies have reported sub-therapeutic effects and associated treatment failure. The current study, therefore, assessed whether persistent schistosomiasis, with its associated morbidity among children living in endemic areas within the Greater Accra Region of Ghana, is as a result of reinfection or suspected praziquantel resistance. Methodology: this was a longitudinal study involving a baseline and follow-up sampling after praziquantel treatment. Urine samples were collected from school children (whose parents had also consented) for the detection of *S. haematobium* ova using a sedimentation technique. The morbidity parameters were examined with urine chemistry strips, as well as microscopy. Viability was assessed using a modified hatchability technique, vital staining (0.4% trypan blue and 1% neutral red) and fluorescent (Hoechst 33258) microscopy. Infected individuals were treated with a single dose of praziquantel (40mg/kg). Resampling to determine reinfection was done sixth months post-treatment, after evidence of total egg clearance. For possible resistance assessment, egg counts and viability testing were conducted on the positive samples at the baseline, as well as weekly post-treatment follow-ups for 12 weeks. Results: out of the 420 school children sampled, 77 were initially positive but, after the sixth month sampling for reinfection assessment, eight out of the initial positives were infected again, giving a reinfection percentage of 10.4%. No suspected praziquantel resistance was recorded in the 21 positives detected out of the 360 sampled for suspected resistance assessment. The egg reduction rate increased weekly in the follow-up samples with a gradual reduction in the egg count. The study also recorded a gradual decrease in the percentage of live eggs after the first week; with all viability testing methods used complimenting each other. The morbidity parameters (proteinuria, haematuria and pyuria) changed between the baseline and post-treatment samples, eventually reducing to zero. Conclusions: the outcome of this study suggests that the persistent schistosomiasis, with its associated morbidity observed in these endemic communities, is not likely to be as a result of praziquantel resistance, but reinfection. Even though there was no suspected resistance observed in the study, there remains the need to continuously intensify the monitoring of praziquantel in other endemic communities.

## 1. Introduction

Schistosomiasis is a neglected tropical disease caused by schistosome species [1]. *Schistosoma haematobium* is the etiologic agent of urogenital schistosomiasis commonly found in Africa, the Middle East and Southern Europe [2]. The geographical distribution of this disease and its transmission zone is closely related to fresh water intermediate host snails such as the *Bulinus* and *Physopsis species* [3,4]. This parasitic infection is usually associated with the lifestyle and behaviour of children especially, such as swimming in infected water bodies [5].

Urogenital schistosomiasis is known to cause haematuria, dysuria, nutritional deficiencies, the risk of bladder cancers and growth retardation in children of school age [5,6]. Haematuria is known to be the main morbidity marker for the diagnosis of urogenital schistosomiasis. Microhaematuria has frequently been identified with urogenital schistosomiasis compared with macrohaematuria, which is a visible sign during the severe stages of this disease. Millions of people are at risk of infection with urogenital schistosomiasis in sub-Saharan Africa [7], with Ghana and neighbouring Nigeria not being an exception. The hyperendemicity of urogenital schistosomiasis has been reported among school children in the Guma Local Government Area, Nigeria [8]. 

Chemotherapy has been one of the most effective means of combating schistosomiasis [9]. Currently, praziquantel remains the number one drug of choice for the treatment of schistosomiasis [10]. The mechanism of action of praziquantel is not well understood. Nevertheless, praziquantel is known to have effects on adult forms of schistosomes which include spastic paralysis of the parasite musculature, possibly through an influx of Ca^2+^ into the worm, as well as vacuolation and degeneration of the worm [11]. Praziquantel has proved to be efficacious against schistosomes when administered orally as a single dose or as a series of double doses, depending on the endemicity within an area [12]. 

The failure of standard treatment with praziquantel was observed in two returning travellers to Spain, who came from Mali and Senegal, with genitourinary schistosomiasis caused by *S. haematobium*, even after repeated doses [13]. Morphologically viable eggs of *S. haematobium* were found in Brazilian military men (who were part of a United Nation peace mission in Mozambique), treated with praziquantel even between six and twenty-four-months post-treatment [14]. A study conducted on pupils (in three rural communities of Kwara State, Nigeria) to determine the reinfection patterns within a year, revealed that 68% of the pupils were reinfected after two doses of praziquantel were administered [15]. Although there is continuous mass administration of praziquantel in schistosomiasis endemic communities in Ghana, there is a dearth of data on the efficacy and/or resistance pattern of the drug. 

Furthermore, there could be sub-therapeutic effects of praziquantel, which may lead to possible praziquantel resistance, as has been reported in other African countries [16,17]. Indeed, there have been reports on the effect of praziquantel treatment on the viability of the eggs of *S. haematobium* [18,19,20]. 

Richards et al. [18] observed that praziquantel kills most *S. mansoni eggs* in host tissues when administered in higher doses. In another study, Matsuda et al. [19] examined the mode of action of praziquantel on *Schistosoma japonicum* eggs in mice and in vitro. Between 1 and 56 days after oral administration of 100 mg/kg four times in one day, they observed many empty egg shells the next day. In their in vitro experiment, eggs from cut pieces of intestine began to hatch starting 5 min after exposure to praziquantel (at a concentration of at least 1 ng/mL), reaching maximum hatching at 30 min, and died several minutes after hatching. Elfaki and colleagues [20] also observed in their study that the mean number of viable eggs in *S. haematobium*-treated patients was significantly lower than the mean number of viable eggs in untreated patients. 

Nonetheless, data on the effectiveness of praziquantel remains crucial, since it is the current drug of choice for the treatment of schistosomiasis and the importance of monitoring the efficacy of the drug cannot be overemphasised. This study is, therefore, aimed at assessing whether persistent schistosomiasis, with its associated morbidity among children living in endemic areas within the Greater Accra Region of Ghana, is as a result of reinfection or suspected praziquantel resistance.

## 2. Materials and Methods 

### 2.1. Design and Study Site 

**Figure 1:**
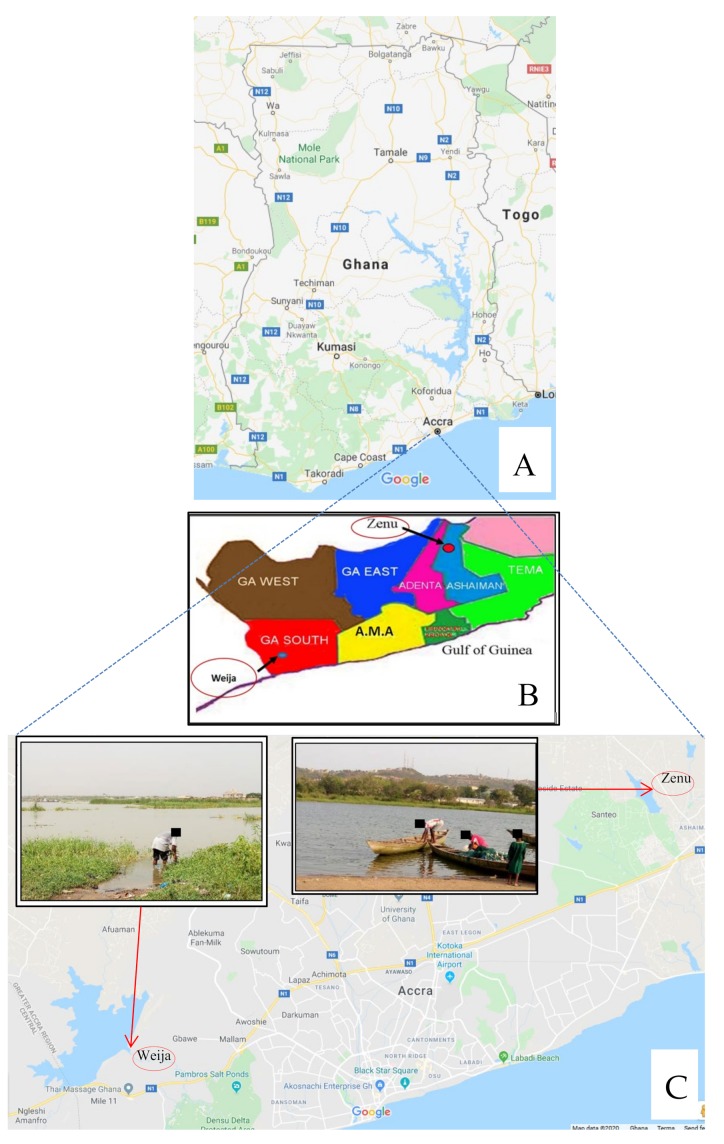
Maps of the geographical area (including sampling sites, lakes and rivers). (**A**) A picture showing the entire map of Ghana; (**B**) a picture showing the Municipal and District Assemblies where the study sites are found; (**C**) a picture showing an extrapolation of part of the greater Accra region with the sampling sites and the presence of water bodies. The inserts in C show some of the human activities in the lakes. The maps are not drawn to scale. A and C were adapted and modified from Google map.com/Greater Accra/Ghana while B was adapted and modified from https://en.wikipedia.org/wiki/Tema_Metropolis_District. Date Accessed: 23/01/2020.

The study was conducted from September, 2016 to March, 2017 among children living in the Zenu and Weija communities of the Greater Accra Region of Ghana. Zenu is a small community situated specifically in the southern part of the Ashiaman municipality (a town on the outskirts of Tema) which is a suburb of Accra, the capital city of Ghana. Its geographical coordinates are 5°42’0” North, 0°20’0” West [21]. Subsistent farming and fishing remain the main economic activities, although a few inhabitants are civil servants (Figure 1). The presence of a lake in the community might have attracted most of the current settlers (Figure 1). The lake serves as a source of drinking water, a place for washing, bathing and other domestic purposes (Figure 1C-inserts). Since children are most often vulnerable and affected by schistosome infection, it became imperative to screen them for this infection. The Zenu community is a “virgin” area in terms of schistosomiasis drug administration by the Neglected Tropical Disease Control Program. The prevalence of urinary schistosomiasis in this community has initially been reported as 30.7% [21]. 

Weija is a community that lies at the South Western part of Accra. There are two main rivers found in this community, namely the Ponpon and the Densu River. The Densu is one of the main sources of water supply to more than half of the population of the Accra Metropolis (Figure 1C). There are several small lakes created by these rivers in the community. The decision to choose Weija for this study was due to the fact that a number of studies had been conducted there [22,23,24], and there is evidence of consistent drug administration by the Neglected Tropical Disease Control Program. The prevalence of urinary schistosomiasis in Weija (Mahem) has initially been reported as 49% [23]. 

The design was a longitudinal study that involved taking baseline samples and subsequent follow-up samples. Approval for this study was granted by the Ethics and Protocol Committee of the College of Health Sciences, University of Ghana (Protocol Identification Number: CHS-Et/M.3 = P 3.5/2016-2017). Urine samples were collected from school children who had assented, and had obtained parental/guardian consent, as a baseline to identify those infected with *S. haematobium*. In assessing reinfection, resampling of previously positive/infected participants was done after sixth months post-praziquantel treatment, to identify those among the previously positive/infected individuals with *S. haematobium* ova in their urine who showed evidence of total egg clearance by the sixth week after treatment (even though, in this study, subsequent checking was done at 8, 10 and 12 weeks). For the assessment of possible resistance, school children with *S. haematobium* eggs in their urine, after an egg count and viability testing (at the baseline), were given praziquantel. These procedures (the egg count and viability testing) were repeated for weekly post-treatment follow-ups until the twelfth week.

### 2.2. Sample Collection, Determination of Morbidity Parameters and Treatment of Infected Participants 

Urine samples were collected from (420 in the reinfection assessment and 360 in the suspected resistance assessment) school children. The samples were collected between the hours of 10:00 am and 12:00 pm for the maximum yield [25]. The urine samples were transported on ice to the Parasitology Laboratory of the Medical Microbiology Department, University of Ghana, for investigation. 

Before the urine samples were examined microscopically, macroscopy was done to determine the colour and appearance of the urine. This was followed by the use of the urine reagent test strips (URIT 10V, URIT Medical Electronic Co., Ltd., Guilin, GZAR, China) to determine parameters such as haematuria, proteinuria, leucocytes, as well as pH, specific gravity, glucose, ketones, bilirubin, and urobilinogen. The collected urine samples were then examined microscopically by aliquoting 10 mL of the urine into a centrifuge tube. These samples were then centrifuged at 3000 rpm for 5 min and supernatant discarded until the 0.5 mL mark. Fifty microlitres (50 µL) of the sediment was transferred onto clean glass slides. This was to examine *S. haematobium* ova which is described by its characteristic oval shape and its terminal spine. 

The egg count was determined by the number of eggs present/10 mL of urine [26]. Individuals who were positive for *S. haematobium* were treated with a single dose of praziquantel by healthcare professionals at Kasoa Polyclinic for children in the Weija community, and Zenu Community Hospital for children in the Zenu community.

### 2.3. Assessment of Reinfection

In assessing reinfection, resampling (of the previously positive participants out of the 420 sampled) was done after sixth months post-praziquantel treatment, to identify individuals with *S. haematobium* ova in their urine who previously showed evidence of total egg clearance by the sixth week after treatment (even though, in this study, subsequent checking was done at 8, 10 and 12 weeks). Reinfection can be described as total egg clearance by the sixth week and the reappearance of eggs (ova) by the sixth month. Therefore, in this study, when the egg counts reduced to zero by the sixth week (and remained at zero even after subsequent checking up to the twelfth week) and eggs then resurfaced in the sixth month, reinfection was said to have likely occurred. 

### 2.4. Assessment of Suspected Resistance

To investigate suspected resistance, school children with *S. haematobium* eggs in their urine (out of the 360 sampled), after egg count and viability testing (at the baseline), were given praziquantel. These procedures (egg count and viability testing) were repeated for weekly post-treatment follow-ups until the twelfth week. Therefore, resistance was suspected when there was evidence of persistent viable eggs (ova) by the sixth week after praziquantel treatment, which still did not clear by the twelfth week. Consequently, if resistance were suspected, viable eggs were expected to be persistently present, up to the sixth and even the twelfth week, without clearing. In terms of egg viability, it had been expected that more live eggs would be found than dead eggs, even after the praziquantel treatment. 

### 2.5. Egg Viability Tests

The viability of the eggs was assessed by modified hatchability, vital and fluorescence staining. For the modified hatchability, the movement of flame cells or the embryo within the egg shell and the swimming of the miracidia as it hatched out of the shell (in the presence of optimum conditions such as water and light) were looked out for [27]. Eggs with these characteristics were considered live/viable, while those with no such movements were considered dead. Viability assessment using vital and fluorescence staining was adapted from our earlier study, which showed the ability of vital and fluorescent staining to differentiate between the live and dead eggs of *Schistosoma haematobium* [28]. 

### 2.6. Analyses of Data 

The data was analyzed using GraphPad Prism version 501. The egg counts between the baseline and follow-ups were used to calculate the arithmetic means of the baseline and post-treatment, which was eventually used to calculate the egg reduction rate (ERR). The statistical difference between the timepoints was tested using ANOVA. A Chi-square (*X^2^*) test was used as a comparative analysis of the methods used to determine viability. *P*-value < 0.05 was considered statistically significant.

## 3. Results

### 3.1. Reinfection or Suspected Resistance 

**Figure 2:**
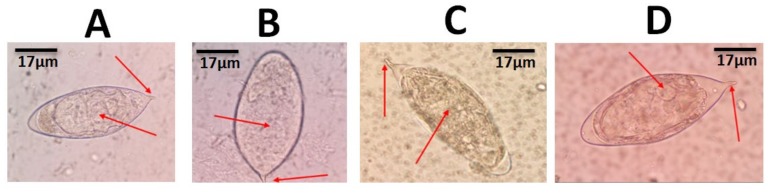
Morphological observations made in shapes and terminal spines of *Schistosoma haematobium* ova during wet mount microscopy using x40 objective lens. (**A** and **B**) The characteristic oval-shaped *S. haematobium* ova with short curved terminal spines; (**C**) a slightly elongated oval-shaped *S. haematobium* ovum with a pointed terminal spine. (**D**) An oval-shaped *S. haematobium* ovum with a slightly curved terminal spine and an intermediate size.

**Figure 3:**
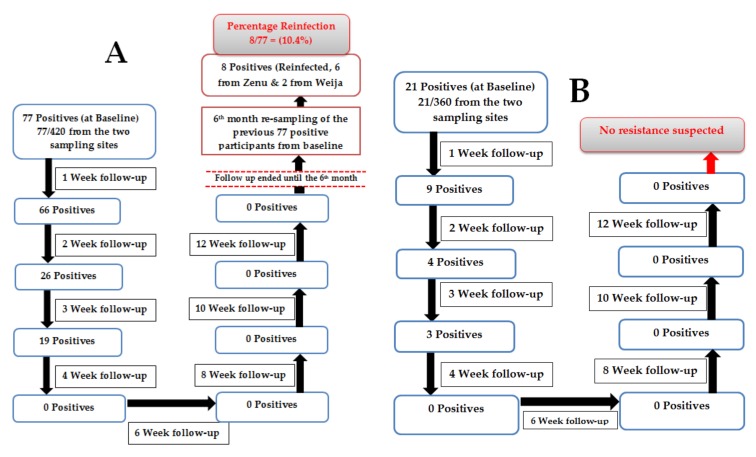
Overview of steps involved in reinfection and suspected resistance assessment. (**A**) Reinfection assessment; (**B**) suspected resistance assessment.

For assessment of reinfection, *Schistosoma haematobium* ova were microscopically detected in 77 out of the 420 school children sampled (from both study areas) at the baseline. Some of these ova are shown in Figure 2. These positive participants gradually reduced in the follow-up to the third week (in week 1, there were 66 positives; in week 2, there were 26 positives; in week 3, there were 19 positives) after which there was no detectable *S. haematobium* ova from the fourth to the twelfth week (Figure 3A). After the six-month sampling of the 77 initial positives, eight participants (six from Zenu and two from Weija) were reinfected, giving a total reinfection percentage of 10.4% (Figure 3A). In the current study, there was no statistically significant difference (*p* = 0.541) between the reinfection percentage for Zenu (10.0%, 6/60) and Weija (11.8%, 2/17).

In the assessment to suspect resistance, 21 out of the 360 participants sampled (from both study areas) at the baseline were positive (Figure 3B). However, there was a reduction in the number of infected participants followed up after praziquantel treatment until the fourth week (Figure 3B), where no positive was recorded (i.e., in week 1, there were 9 positives; in week 2, there were 4 positives; in week 3, there were 3 positives; in week 4, there were no positives). Subsequent follow-ups from the sixth week to the twelfth week showed no evidence of *S. haematobium* ova in any of the study participants, indicating egg clearance (Figure 3B). An overview of the steps involved in reinfection and suspected resistance assessment is shown in Figure 3.

### 3.2. Mean Egg Count and Egg Reduction Rates

**Figure 4:**
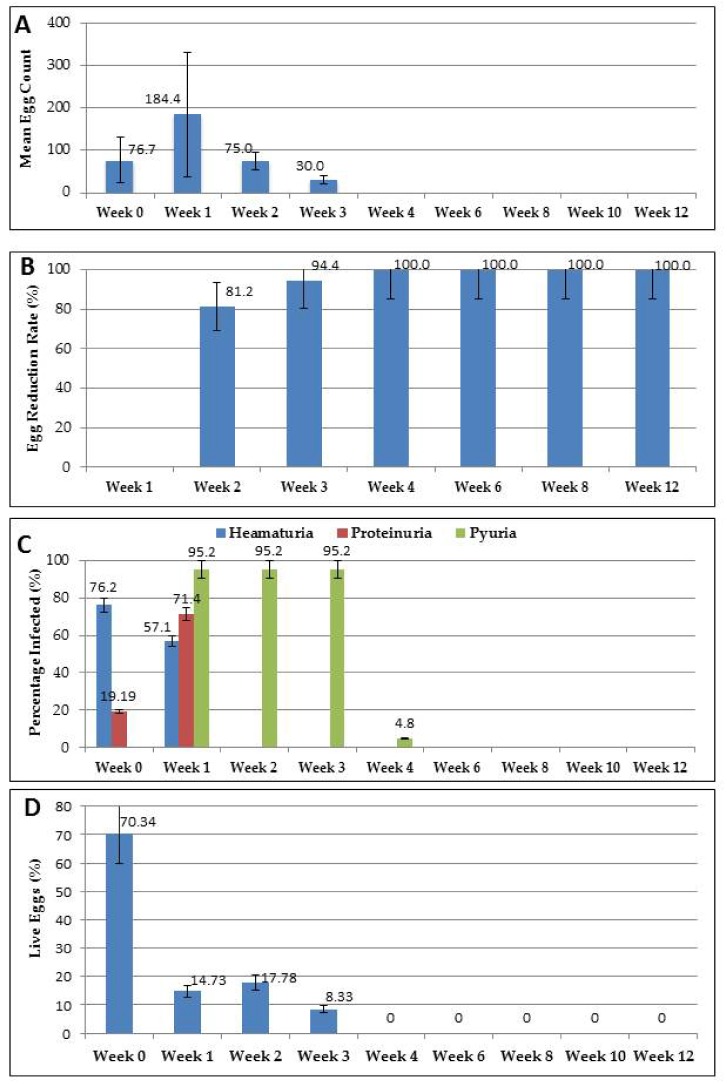
Egg counts and morbidity parameters. (**A**) The mean egg count; (**B**) the egg reduction rates; (**C**) the morbidity parameters; (**D**) the viability percentage. No eggs were detected from week 4 to week 12. The error bars represent standard deviation.

The mean egg count (MEC) recorded at the baseline for the 21 positives out of the 360 participants sampled for the suspected resistance assessment was 76.67 eggs per 10mL [95% CI: 52.18, 101.15]. This was slightly lower than the mean egg count of the first week (MEC = 184.44 eggs per 10mL [95% CI: 124.26, 244.62]) of the follow-up (post-treatment). From the second week of the follow-up, there was a reduction in the mean egg count (week 2 = 75.00 [95% CI: 60.78, 89.22] and week 3 = 30.00 [95% CI: 24.89, 35.10]), until the fourth week, where no eggs were detected, even up to the twelfth week (Figure 4A, see Supplementary Table S1). Similarly, the egg reduction rates (ERR) at week 2 and week 3 were 81.24% and 94.37%, respectively (Figure 4B) while those of the fourth, sixth, eighth, as well as the twelfth week remained at 100% (Figure 4B). Among the examination periods, the difference in the mean egg counts (*p =* 0.321) and the egg reduction rates (*p* = 0.126) were not statistically significant.

### 3.3. Assessment of Morbidity Parameters

There were differences in the morbidity parameters (such as haematuria, proteinuria and pyuria) between the baseline and the weekly follow-ups (Figure 4C). Haematuria and proteinuria were detected at the baseline, while pyuria was not (Figure 4C). Among the infected participants, 76.2% [95% CI: 65.1, 87.3] had haematuria at the baseline, 57.1% [95% CI: 46.2, 68.0] at week 1 and 0% had it in subsequent weeks. Proteinuria was lower at the baseline (19.2% [95% CI: 11.4, 27.0]) compared to the first week (71.4% [95% CI: 62.4, 80.4]), and was also undetectable in the subsequent follow-ups (0.0%). In the case of pyuria, which was not detected at the baseline, there were higher stable detectable levels of it during the first and up to the third week post-treatment (week 1 = 95.2% [95% CI: 84.3, 105.7], week 2 = 95.2% [95% CI: 84.3, 105.7], week 3 = 95.2% [95% CI: 84.3, 105.7]) and low levels in the fourth week (4.8%), becoming undetectable in the subsequent weeks (Figure 4C). There were no statistically significant differences in the morbidity parameters among the various weeks (*p* = 0.3822).

### 3.4. Viability at Baseline and Post-Treatment

**Figure 5:**
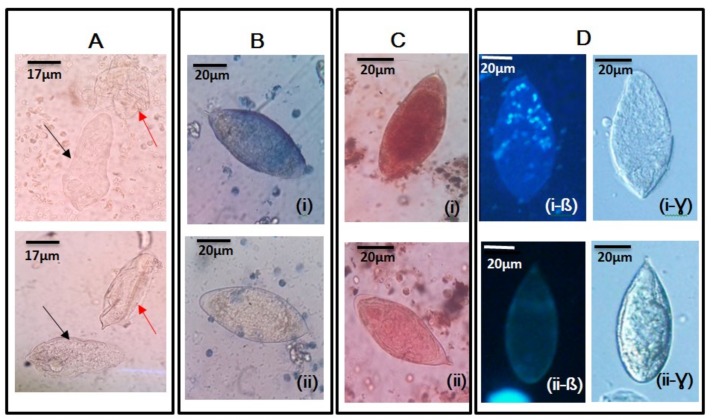
Egg viability testing methods (modified hatchability, vital and fluorescence staining of *Schistosoma haematobium* eggs. (**A**) The modified hatchability test with black arrows showing the miracidia that have moved out of the egg shells, indicated with red arrows. (**B**) *S. haematobium* eggs stained by 0.4% trypan blue observed at ×40 through an objective lens: (i) shows an egg that retained the 0.4% trypan blue, indicative of a dead *S. haematobium* egg, while (ii) shows an egg that did not retain the 0.4% trypan blue stain, indicative of a live egg. (**C**) *S. haematobium* eggs stained with 1% neutral red and observed at ×40 through an objective lens: (i) shows an egg with retention of 1% the neutral red, indicative of a live egg, while (ii) shows an egg with non-retention of 1% the neutral red stain, indicative of a dead egg. (**D**) *Schistosoma haematobium* ova observed using fluorescent microscopy, with the cell stain (Hoechst 33258). (i-ß) An egg showing fluorescence (blue), indicative of a dead egg, with i-Ɣ being a corresponding light microscopy picture. (ii-ß) An egg demonstrating no fluorescence, indicative of a live egg, with ii-Ɣ being a corresponding light microscopy picture.

The proportion of live eggs to dead eggs at the baseline was higher compared to that of post- treatment (Table 1), considering all the methods (the modified hatchability technique, the vital staining and the fluorescent microscopy) used to determine viability (Figure 4, see Supplementary Tables S2–S5). All methods used to determine the viability of *S. haematobium* ova in the study produced similar outcomes and, thus, they complemented/supported each other. There was no statistically significant difference in the outcome for the different methods (*X^2^* = 9.863, *p* = 0.101) (Table 1, Figure 5). The mean percentage of live eggs and dead eggs at the baseline was 70.34% [95% CI: 57.42, 83.26] and 29.66% [95% CI: 16.74, 42.57], respectively (Table 1). However, 13.57% [95% CI: 11.52, 15.62] live eggs and 86.73% [95% CI: 84.38, 88.48] dead eggs were recorded at post-treatment. The difference between the amount of live eggs and dead eggs at the baseline compared to what was recorded post-treatment was statistically significant (*p* < 0.00001). The percentage of live eggs decreased to below 20% from week 1 to week 3 (week 1 = 14.73%, week 2 = 17.78% and week 3 = 8.33%) (Figure 4D, Table 1), with no eggs (live or dead) detected from week 4 to week 12 (Figure 4D). 

## 4. Discussion

The findings of this study, which indicated a total reinfection percentage of 10.4% from the two sites suggests that school children living in endemic areas could still be exposed to *S. haematobium* infection, even after praziquantel treatment. This reinfection percentage (10.4%) corroborates similar work done by Senghor et al. [29] and Kabuyaya et al. [30], who also reported reinfection rates of 12.6% and 8.1%, respectively. Woldegerima et al. [31] also reported a reinfection rate of 13.9%, after 6 months of post-treatment, although this was in *S. mansoni*. Meanwhile, a high reinfection percentage of 58% was reported by Oniya and Odaibo [15]. Studies has shown that, low reinfection percentage of *S. haematobium* can be attributed to the effectiveness of the treatment, particularly in school-age children [32,33], and the season in which sampling was done [30,34]. 

In the study by Kabuyaya et al. [30], the low reinfection percentage observed was associated with the persistent drought throughout their study period. Also, Midzi et al. [34] showed that a considerable number of the transmission hotspots (water sources) dried up during their study period, thus limiting the exposure of children to water contact, which might have contributed to the low reinfection percentage. On the other hand, other factors, such as the ecology and being in an area with a high intensity of infection, have been implicated in the high reinfection rates observed in other studies [15,30,35]. 

According to Kabuyaya and colleagues, people might become re-infected when they revert to their previous daily activities, involving contact with water infested with intermediate hosts, after a successful treatment with praziquantel [30]. Similarly, residents in the study sites use the lakes located in the communities for washing and bathing, as well as occupational and other domestic purposes. Therefore, after successful treatment, they needed to revert to these activities involving contact with the lakes, resulting in the reinfection observed in the current study. Therefore, the continuous exposure to water, as well as water contact activities, could probably have contributed to the reinfection observed in this study, as previously indicated by Mostafa et al. [6].

In this study, the assessment of reinfection in the sixth month, (even when there has been evidence of total egg clearance at the fourth, sixth, eighth and twelfth weeks), agrees with a similar study in which reinfection patterns were also monitored among *S. haematobium-*infected individuals, six months after treatment [29]. The pre-patent period (from infection to excretion of eggs by the host) of *S. haematobium* is known to be between 8 to 10 weeks [36], therefore, egg clearance by the twelfth week suggests that there was no evidence of a new infection during the follow-up sampling, although some participants later became reinfected in the sixth month. 

Resistance was not suspected in this study after treatment with praziquantel in the follow-up samples, which suggests that the drug continues to be effective in the treatment of urinary schistosomiasis. This finding agrees with what was observed by Ojurongbe et al. [37] in Nigeria. They also could not detect the presence of viable eggs in the follow-up samples post-praziquantel treatment. Other studies, however, revealed the presence of persistent eggs after praziquantel treatment in the follow-up samples of the study participants, suggesting the possible reduced efficacy of the drug [13,14,30]. 

Parameters such as the egg reduction rate [30,34,38] and viability [33] which had been used by other researchers [29,31,33,37] and were then adapted as tools during the follow-up assessment for suspecting resistance in this study, gave consistent results. The egg reduction rates (ERR) recorded in this study (with the egg count pattern) from the second week to the twelfth week (76.3% to 100%) agrees with a study conducted at Abeokuta in Nigeria with an ERR between 57% and 100% by the twelfth week [37]. This demonstrates, somewhat, the efficacy of praziquantel. In this study, the ERR of 86.5%, observed in the third week post-treatment, was slightly lower compared to a recent report by Woldegerima et al. [31] who observed egg reduction rates of 99.5% in *S. mansoni.* The difference in the species of *Schistosoma* and the study sites between the current study and that of Woldegerima et al. [31] could probably have accounted for the slight difference in the reduction rates. However, the relatively low egg reduction rates, between 64% and 80%, observed in South Africa [30] were attributed to low drug absorption and high levels of catabolism, but not resistance [30]. 

In this study, the viability percentage of the live eggs being lower than 20% in the first, second and third week follow-ups, compared to that of the baseline (70.34%) after treatment, suggest the possible impact of praziquantel treatment on the viability of *S. haematobium* eggs. This observation is similar to a study by Elfaki et al. [20], who reported that the mean number of viable eggs in treated patients was lower than the mean number of viable eggs in untreated patients. 

In this study, the mean egg count at the baseline being higher than that of the subsequent follow-ups agrees with a study conducted in the Cote d’Ivoire, which reported a higher mean egg count of 84.8 eggs/10 mL at the baseline, relative to a range of 40.5 to 0.3 eggs/10mL in the post-treatment phase [39]. However, the observation of eggs shed even after weeks of treatment in this study does not imply that the praziquantel treatment was ineffective and, thus, clinicians should not be very worried if eggs are detected in the laboratory, even after they have treated the patients. Additionally, some of these eggs might not even be viable, hence, adapting the viability testing in addition to the egg reduction rates could be useful in monitoring the effect of praziquantel on schistosomiasis control. 

In the current study, the morbidity parameters, such as haematuria, proteinuria and pyuria (leucocytes) were associated with *S. haematobium* infection, and this agrees with work done by Gryseels et al. (2006) [1], Stete et al. [39], Mekonnen et al. [40] and Wami et al. [41], who also reported the association between haematuria, proteinuria and pyuria with urogenital schistosomiasis. The percentage increase in proteinuria (from 19.19% to 71.4%) and the decrease in haematuria (from 76.2% to 57.1%) in follow-up samples could be attributed to the activity of praziquantel in the body. This observation is slightly different to earlier studies in which proteinuria and haematuria were reduced, from 88% to 16% and 73% to 4% [39], as well as from 100% to 40% and 94.1% to 48.7% [40], respectively. The reduction in leucocytes (detected only in the urine of post-treatment samples) from 95.2% in the first and second weeks to 4.8% in the third week, agrees with a study conducted in the Cote D’Ivoire which reported 71% leucocytes, which reduced to 2% in the 5 weeks post-treatment [39]. The high number of leucocytes in the urine in the first week after treatment has been found to be as a result of inflammation and lesions in the urinary tract during the drug’s response to the infection [42]. The non-detection of proteinuria, haematuria and pyuria 4 weeks post-praziquantel treatment can be indirectly attributed to the effectiveness of the treatment. 

The higher mean viability percentage at the baseline, relative to post-treatment, suggests some of the effects of praziquantel on *S. haematobium* ova. This finding is similar to work done in Sudan [20] on evaluating the effect of praziquantel treatment on the eggs of *S. haematobium*. Their study revealed more viable eggs in the baseline samples when compared to the post-treatment samples, and more non-viable eggs in the post-treatment samples when compared to the baseline samples [20]. 

The modified hatchability technique was a good tool for determining the live and dead eggs of *S. haematobium*, as a result of the visualisation of the presence of flame cells and movement in the embryo. This technique complements the fluorescent dye (Hoechst 33258), as well as the vital stains, which have been reported as having the ability to differentiate between live and dead eggs [27].

## 5. Conclusions

The current study showed a reinfection percentage of 10.4%, but no suspected resistance to praziquantel. A gradual decrease in the percentage of live eggs after the first week was also recorded in the study, with all viability testing methods used complimenting each other. The egg reduction rate increased weekly in the follow-up samples, with a gradual reduction in the egg count. The morbidity parameters (proteinuria, haematuria and pyuria) changed between the baseline and post-treatment samples, eventually reducing to zero. The outcome of this study suggests that persistent schistosomiasis, with its associated morbidity observed in these endemic communities, is not likely to be as a result of praziquantel resistance, but reinfection. Even though there was no suspected resistance observed in this study, there is the need to continuously intensify the monitoring of praziquantel in other endemic communities.

## Figures and Tables

**Table 1:** Viability at baseline and post-treatment.

Method Used #	Baseline (%) (Pre-Treatment)		Post-Treatment (%)							
			Week 1		Week 2		Week 3		Average *	
	Live	Dead	Live	Dead	Live	Dead	Live	Dead	Live	Dead
**Trypan blue**	60.25	39.75	19.16	80.84	27.27	72.27	0.00	100.00	15.47	84.53
**Neutral red**	70.81	29.19	12.05	87.95	16.70	83.33	11.11	88.89	13.28	86.72
**Fluorescent microscopy**	80.12	19.88	13.86	86.14	13.33	86.67	11.11	88.89	12.76	87.24
**Modified hatchability**	70.19	29.81	13.86	86.14	13.33	86.67	11.11	88.89	12.77	87.23
**Mean**	70.34	29.66	14.73	85.27	17.78	82.09	8.33	91.67	13.57	86.43

* Average = (week 1 + week 2 + week 3)/3, since there were no eggs detected from the fourth week to the twelfth week. # There was no statistically significant difference in the outcome for the different methods used (X^2^ = 9.863, *p* = 0.101).

## Supplementary Materials

The following are available online at https://www.mdpi.com/2076-3271/8/1/10/s1, Table S1: Egg count (eggs per 10ml) recorded at baseline and post-treatment for the 21 positive participants, Table S2: Proportions of live and dead eggs for Trypan blue staining, Table S3: Proportions of live and dead eggs for Neutral red staining, Table S4: Proportions of live and dead eggs for Fluorescent staining, Table S5: Proportions of live and dead eggs for Modified Hatchability Test.
