# Supplementary material for: Persistent Urogenital Schistosomiasis and Its Associated Morbidity in Endemic Communities within Southern Ghana: Suspected Praziquantel Resistance or Reinfection?
Source: Med Sci (Basel). 2020 Feb 10;8(1):10. doi: 10.3390/medsci8010010 (PMC7151560; doi:10.3390/medsci8010010)
Supplement: Supplementary file 1 [file medsci-08-00010-s001.pdf]

Supplementary Materials

Table S1: Egg count (eggs per 10ml) recorded at baseline and post-treatment for the 21 positive participants

Sample Number	Week 0	Week 1	Week 2	Week 3	Week 4	Week 6	Week 8	Week 10	Week 12
1	110	330	0	0	0	0	0	0	0
2	140	0	0	0	0	0	0	0	0
3	30	80	0	0	0	0	0	0	0
4	150	220	50	0	0	0	0	0	0
15	60	0	0	0	0	0	0	0	0
6	30	20	0	0	0	0	0	0	0
7	20	0	0	0	0	0	0	0	0
8	100	130	80	40	0	0	0	0	0
9	30	0	0	0	0	0	0	0	0
10	40	20	0	0	0	0	0	0	0
11	130	0	0	0	0	0	0	0	0
12	80	0	0	0	0	0	0	0	0
13	60	0	0	0	0	0	0	0	0
14	10	0	0	0	0	0	0	0	0
15	180	320	70	30	0	0	0	0	0
16	40	0	0	0	0	0	0	0	0
17	20	0	0	0	0	0	0	0	0
18	40	0	0	0	0	0	0	0	0
9	50	110	0	0	0	0	0	0	0
20	120	430	100	20	0	0	0	0	0
21	170	0	0	0	0	0	0	0	0
Total (Eggs per 10ml)	1610	1660	300	90	0	0	0	0	0
Mean (Eggs per 10ml)	76.66667	184.4444	75	30	0	0	0	0	0
SD	53.78971	147.7423	20.81666	10	0	0	0	0	0

SD: Standard Deviation

Table S2: Proportions of live and dead eggs for Trypan blue staining

Sample Number	Week 0 (Baseline)		Week 1		Week 2		Week 3		Week 4 - Week 12	
	Live, n (%)	Dead, n (%)	Live, n (%)	Dead, n (%)	Live, n (%)	Dead, n (%)	Live, n (%)	Dead, n (%)	Live, n (%)	Dead, n (%)
**1**	80 (72.73)	30 (27.27)	60 (18.18)	270 (81.82)	0 (0)	0 (0.00)	0(0)	0	0(0)	0(0)
**2**	80 (57.14)	60 (42.86)	0 (0)	0 (0)	0 (0)	0 (0.00)	0(0)	0	0(0)	0(0)
**3**	20 (66.67)	10 (33.33)	30 (37.5)	50 (62.5)	0 (0)	0 (0.00)	0(0)	0	0(0)	0(0)
**4**	110 (73.33)	40 (26.67)	60 (27.27)	160 (72.73)	10 (20)	40 (80.00)	0(0)	0	0(0)	0(0)
**5**	30 (50)	30 (50)	0 (0)	0 (0)	0 (0)	0 (0.00)	0(0)	0	0(0)	0(0)
**6**	10 (33.33)	20 (66.67)	0 (0)	20 (100)	0 (0)	0 (0.00)	0(0)	0	0(0)	0(0)
**7**	10 (50)	10 (50)	0 (0)	0 (0)	0 (0)	0 (0.00)	0(0)	0	0(0)	0(0)
**8**	60 (60)	40 (40)	40 (28.57)	100 (71.43)	0 (0)	90 (100)	0(0)	40 (100)	0(0)	0(0)
**9**	20 (66.67)	10 (33.33)	0 (0)	0 (0)	0 (0)	0 (0.00)	0(0)	0	0(0)	0(0)
**10**	20 (50)	20 (50)	0 (0)	20 (100)	0 (0)	0 (0.00)	0(0)	0	0(0)	0(0)
**11**	70 (53.85)	60 (46.15)	0 (0)	0 (0)	0 (0)	0 (0.00)	0(0)	0	0(0)	0(0)
**12**	40 (50)	40 (50)	0 (0)	0 (0)	0 (0)	0 (0.00)	0(0)	0	0(0)	0(0)
**13**	50 (83.33)	10 (16.67)	0 (0)	0 (0)	0 (0)	0 (0.00)	0(0)	0	0(0)	0(0)
**14**	10 (100)	0 (0)	0 (0)	0 (0)	0 (0)	0 (0.00)	0(0)	0	0(0)	0(0)
**15**	80 (44.44)	100(55.56)	70 (21.88)	250 (78.13)	30 (42.86)	40 (57.14)	0(0)	30 (100)	0(0)	0(0)
**16**	20 (50)	20 (50)	0 (0)	0 (0)	0 (0)	0 (0.00)	0(0)	0	0(0)	0(0)
**17**	10 (50)	10 (50)	0 (0)	0 (0)	0 (0)	0 (0.00)	0(0)	0	0(0)	0(0)
**18**	30 (75)	10 (25)	0 (0)	0 (0)	0 (0)	0 (0.00)	0(0)	0	0(0)	0(0)
**19**	40 (80)	10 (20)	20 (18.18)	90 (81.82)	0 (0)	0 (0.00)	0(0)	0	0(0)	0(0)
**20**	70 (58.33)	50 (41.67)	40 (9.30)	390 (90.7)	50 (41.67)	70 (58.33)	0(0)	20 (100)	0(0)	0(0)
**21**	110 (64.71)	60 (35.29)	0 (0)	0 (0)	0 (0)	0 (0.00)	0(0)	0	0(0)	0(0)
**9**	40 (80)	10 (20)	20 (18.18)	90 (81.82)	0 (0)	0 (0.00)	0(0)	0	0(0)	0(0)
Total	970 (60.25)	640(39.75)	320 (19.16)	1350 (80.84)	90 (27.27)	240 (72.73)	0(0)	90 (100)	0(0)	0(0)
Mean	46.19	30.476	35.56	150	22.50	60	0(0)	30	0(0)	0(0)
SD	32.78	24.794	25.55	128.5	20	24.49	0(0)	10	0(0)	0(0)

SD: Standard Deviation

Table S3: Proportions of live and dead eggs for Neutral red staining

Sample Number	Week 0 (Baseline)		Week 1		Week 2		Week 3		Week 4 - Week 12	
	Live, n (%)	Dead, n (%)	Live, n (%)	Dead, n (%)	Live, n (%)	Dead, n (%)	Live, n (%)	Dead, n (%)	Live, n (%)	Dead, n (%)
**1**	80 (72.73)	30 (27.27)	40 (12.12)	290(87.88)	0 (0.00)	0 (0.00)	0 (0.00)	0 (0.00)	0(0.00)	0(0.00)
**2**	110 (78.57)	30 (21.43)	0 (0.00)	0 (0.00)	0 (0.00)	0 (0.00)	0 (0.00)	0 (0.00)	0(0.00)	0(0.00)
**3**	20 (66.67)	10 (33.33)	10 (12.50)	70 (87.50)	0 (0.00)	0 (0.00)	0 (0.00)	0 (0.00)	0(0.00)	0(0.00)
**4**	110 (73.33)	40 (26.67)	30(13.64)	190(86.36)	10 (20.00)	40(80.00)	0 (0.00)	0 (0.00)	0(0.00)	0(0.00)
**5**	30 (50.00)	30 (50.00)	0 (0.00)	0 (0.00)	0 (0.00)	0 (0.00)	0 (0.00)	0 (0.00)	0(0.00)	0(0.00)
**6**	10 (33.33)	20 (66.67)	0 (0.00)	20 (100.00)	0 (0.00)	0 (0.00)	0 (0.00)	0 (0.00)	0(0.00)	0(0.00)
**7**	10 (50.00)	10 (50.00)	0 (0.00)	0 (0.00)	0 (0.00)	0 (0.00)	0 (0.00)	0 (0.00)	0(0.00)	0(0.00)
**8**	60 (60.00)	40 (40.00)	20(15.38)	110(84.62)	30 (30.00)	70(70.00)	10 (25.00)	30(75.00)	0(0.00)	0(0.00)
**9**	20 (66.67)	10 (33.33)	0 (0.00)	0 (0.00)	0 (0.00)	0 (0.00)	0 (0.00)	0(0.00)	0(0.00)	0(0.00)
**10**	20 (50.00)	20 (50.00)	0 (0.00)	20 (100.00)	0 (0.00)	0 (0.00)	0 (0.00)	0(0.00)	0(0.00)	0(0.00)
**11**	90 (69.23)	40 (30.77)	0 (0.00)	0(0.00)	0 (0.00)	0 (0.00)	0 (0.00)	0(0.00)	0(0.00)	0(0.00)
**12**	40 (50.00)	40 (50.00)	0 (0.00)	0 (0.00)	0 (0.00)	0 (0.00)	0 (0.00)	0(0.00)	0(0.00)	0(0.00)
**13**	50 (83.33)	10 (16.67)	0 (0.00)	0 (0.00)	0 (0.00)	0 (0.00)	0 (0.00)	0(0.00)	0(0.00)	0(0.00)
**14**	10 (100.00)	0 (0.00)	0 (0.00)	0 (0.00)	0 (0.00)	0 (0.00)	0 (0.00)	0(0.00)	0(0.00)	0(0.00)
**15**	150 (83.33)	30 (16.67)	30(9.36)	290(90.63)	10 (12.50)	70(87.50)	0 (0.00)	30(100.00)	0(0.00)	0(0.00)
**16**	20 (50.00)	20 (50.00)	0 (0.00)	0 (0.00)	0 (0.00)	0 (0.00)	0 (0.00)	0(0.00)	0(0.00)	0(0.00)
**17**	10 (50.00)	10 (50.00)	0 (0.00)	0 (0.00)	0 (0.00)	0 (0.00)	0 (0.00)	0(0.00)	0(0.00)	0(0.00)
**18**	30 (75.00)	10 (25.00)	0 (0.00)	0 (0.00)	0 (0.00)	0 (0.00)	0 (0.00)	0(0.00)	0(0.00)	0(0.00)
**19**	40 (80.00)	10 (20.00)	20(18.18)	90(81.82)	0 (0.00)	0 (0.00)	0 (0.00)	0(0.00)	0(0.00)	0(0.00)
**20**	70 (58.33)	50 (41.67)	50 (11.63)	380(88.37)	0 (0.00)	70(100.00)	0 (0.00)	20(100.00)	0(0.00)	0(0.00)
**21**	160 (94.12)	10 (5.88)	0 (0.00)	0 (0.00)	0 (0.00)	0 (0.00)	0 (0.00)	0(0.00)	0(0.00)	0(0.00)
Total	1140 (70.81)	470(29.19)	200(12.05)	1460(87.95)	50(16.67)	250(83.33)	10(11.11)	80(88.89)	0(0.00)	0(0.00)
Mean	54.28	22.38	22.222222	162.2	12.5	62.5	3.33	26.67	0	0
SD	46.32	14.11	17.159384	131.2	12.583057	15	5.77	5.774	0	0

SD: Standard Deviation

Table S4: Proportions of live and dead eggs for Fluorescent staining

Sample Number	Week 0 (Baseline)		Week 1		Week 2		Week 3		Week 4 - Week 12	
	**Live, n (%)**	**Dead, n (%)**	**Live, n (%)**	**Dead, n (%)**	**Live, n (%)**	**Dead, n (%)**	**Live, n (%)**	**Dead, n (%)**	**Live, n (%)**	**Dead, n (%)**
**1**	100 (90.91)	10(9.09)	60(18.18)	270(81.82)	0(0.00)	0(0.00)	0(0.00)	0(0.00)	0(0.00)	0(0.00)
**2**	120 (85.71)	20(14.29)	0(0.00)	0(0.00)	0(0.00)	0(0.00)	0(0.00)	0(0.00)	0(0.00)	0(0.00)
**3**	20 (66.67)	10(33.33)	10(12.50)	70(87.50)	0(0.00)	0(0.00)	0(0.00)	0(0.00)	0(0.00)	0(0.00)
**4**	140 (93.33)	10(6.67)	40(18.18)	180(81.82)	0(0.00)	50(100.00)	0(0.00)	0(0.00)	0(0.00)	0(0.00)
**5**	30(50.00)	30(50.00)	0(0.00)	0(0.00)	0(0.00)	0(0.00)	0(0.00)	0(0.00)	0(0.00)	0(0.00)
**6**	10(33.33)	20(66.67)	0(0.00)	20(100.00)	0(0.00)	0(0.00)	0(0.00)	0(0.00)	0(0.00)	0(0.00)
**7**	10(50.00)	10(50.00)	0(0.00)	0(0.00)	0(0.00)	0(0.00)	0(0.00)	0(0.00)	0(0.00)	0(0.00)
**8**	80(80.00)	20(20.00)	10(7.69)	120(92.31)	10(11.11)	80(88.89)	10(25.00)	30(75.00)	0(0.00)	0(0.00)
**9**	20(66.67)	10(33.33)	0(0.00)	0(0.00)	0(0.00)	0(0.00)	0(0.00)	0(0.00)	0(0.00)	0(0.00)
**10**	20(50.00)	20(50.00)	0(0.00)	20(100.00)	0(0.00)	0(0.00)	0(0.00)	0(0.00)	0(0.00)	0(0.00)
**11**	110(84.62)	20(15.38)	0(0.00)	0(0.00)	0(0.00)	0(0.00)	0(0.00)	0(0.00)	0(0.00)	0(0.00)
**12**	60(75.00)	20(25.00)	0(0.00)	0(0.00)	0(0.00)	0(0.00)	0(0.00)	0(0.00)	0(0.00)	0(0.00)
**13**	50(83.33)	10(16.67)	0(0.00)	0(0.00)	0(0.00)	0(0.00)	0(0.00)	0(0.00)	0(0.00)	0(0.00)
**14**	10(100.00)	0(0.00)	0(0.00)	0(0.00)	0(0.00)	0(0.00)	0(0.00)	0(0.00)	0(0.00)	0(0.00)
**15**	160(88.89)	20(11.11)	60(18.75)	260(81.25)	10(14.29)	60(85.71)	0(0.00)	30(100.00)	0(0.00)	0(0.00)
**16**	30(75.00)	10(25.00)	0(0.00)	0(0.00)	0(0.00)	0(0.00)	0(0.00)	0(0.00)	0(0.00)	0(0.00)
**17**	10(50.00)	10(50.00)	0(0.00)	0(0.00)	0(0.00)	0(0.00)	0(0.00)	0(0.00)	0(0.00)	0(0.00)
**18**	30(75.00)	10(25.00)	0(0.00)	0(0.00)	0(0.00)	0(0.00)	0(0.00)	0(0.00)	0(0.00)	0(0.00)
**19**	40(80.00)	10(20.00)	10(9.09)	100(90.91)	0(0.00)	0(0.00)	0(0.00)	0(0.00)	0(0.00)	0(0.00)
**20**	90(75.00)	30(25.00)	40(9.30)	390(90.70)	20(22.22)	70(77.78)	0(0.00)	20(100.00)	0(0.00)	0(0.00)
**21**	150(88.24)	20(11.76)	0(0.00)	0(0.00)	0(0.00)	0(0.00)	0(0.00)	0(0.00)	0(0.00)	0(0.00)
Total	1290(80.12)	320(19.88)	230(13.86)	1430(86.14)	40(13.33)	260(86.67)	10(11.11)	80(88.89)	0(0.00)	0(0.00)
Mean	61.42857143	15.2381	25.55556	143	10	65	3.3333333	26.67	0	0
SD	50.72333923	7.496031	24.55153	126.4362	8.16496581	12.90994	5.7735027	5.774	0	0

SD: Standard Deviation

Table S5: Proportions of live and dead eggs for Modified Hatchability Test

Sample Number	Week 0 (Baseline)		Week 1		Week 2		Week 3		Week 4 - Week 12	
	**Live, n (%)**	**Dead, n (%)**	**Live, n (%)**	**Dead, n (%)**	**Live, n (%)**	**Dead, n (%)**	**Live, n (%)**	**Dead, n (%)**	**Live, n (%)**	**Dead, n (%)**
**1**	80(72.73)	30(27.27)	60(18.18)	270(81.82)	0(0.00)	0(0.00)	0(0.00)	0(0.00)	0(0.00)	0(0.00)
**2**	100(71.43)	40(28.57)	0(0.00)	0(0.00)	0(0.00)	0(0.00)	0(0.00)	0(0.00)	0(0.00)	0(0.00)
**3**	20(66.67)	10(33.33)	10(12.50)	70(87.50)	0(0.00)	0(0.00)	0(0.00)	0(0.00)	0(0.00)	0(0.00)
**4**	110(73.33)	40(26.67)	50(22.73)	170(77.27)	0(0.00	50(100.00)	0(0.00)	0(0.00)	0(0.00)	0(0.00)
**15**	30(50.00)	30(50.00)	0(0.00)	0(0.00)	0(0.00)	0(0.00)	0(0.00)	0(0.00)	0(0.00)	0(0.00)
**6**	10(33.33)	20(66.67)	0(0.00)	20(100.00)	0(0.00)	0(0.00)	0(0.00)	0(0.00)	0(0.00)	0(0.00)
**7**	10(50.00)	10(50.00)	0(0.00)	0(0.00)	0(0.00)	0(0.00)	0(0.00)	0(0.00)	0(0.00)	0(0.00)
**8**	60(60.00)	40(40.00)	20(15.38)	110(84.62)	10(11.11)	80(88.89)	10(25.00)	30(75.00)	0(0.00)	0(0.00)
**9**	20(66.67)	10(33.33)	0(0.00)	0(0.00)	0(0.00)	0(0.00)	0(0.00)	0(0.00)	0(0.00)	0(0.00)
**10**	20(50.00)	20(50.00)	0(0.00)	20(100.00)	0(0.00)	0(0.00)	0(0.00)	0(0.00)	0(0.00)	0(0.00)
**11**	90(69.23)	40(30.77)	0(0.00)	0(0.00)	0(0.00)	0(0.00)	0(0.00)	0(0.00)	0(0.00)	0(0.00)
**12**	40(50.00)	40(50.00)	0(0.00)	0(0.00)	0(0.00)	0(0.00)	0(0.00)	0(0.00)	0(0.00)	0(0.00)
**13**	50(83.33)	10(16.67)	0(0.00)	0(0.00)	0(0.00)	0(0.00)	0(0.00)	0(0.00)	0(0.00)	0(0.00)
**14**	10(100.00)	0(0.00)	0(0.00)	0(0.00)	0(0.00)	0(0.00)	0(0.00)	0(0.00)	0(0.00)	0(0.00)
**15**	150(83.33)	30(16.67)	40(12.50)	280(87.50)	10(14.29)	60(85.71)	0(0.00)	30(100.00)	0(0.00)	0(0.00)
**16**	20(50.00)	20(50.00)	0(0.00)	0(0.00)	0(0.00)	0(0.00)	0(0.00)	0(0.00)	0(0.00)	0(0.00)
**17**	10(50.00)	10(50.00)	0(0.00)	0(0.00)	0(0.00)	0(0.00)	0(0.00)	0(0.00)	0(0.00)	0(0.00)
**18**	30(75.00)	10(25.00)	0(0.00)	0(0.00)	0(0.00)	0(0.00)	0(0.00)	0(0.00)	0(0.00)	0(0.00)
**19**	40(80.00)	10(20.00)	10(9.09)	100(90.91)	0(0.00)	0(0.00)	0(0.00)	0(0.00)	0(0.00)	0(0.00)
**20**	70(58.33)	50(41.67)	40(9.30)	390(90.70)	20(22.22)	70(77.78)	0(0.00)	20(100.00)	0(0.00)	0(0.00)
**21**	160(94.12)	10(5.88)	0(0.00)	0(0.00)	0(0.00)	0(0.00)	0(0.00)	0(0.00)	0(0.00)	0(0.00)
Total	1130(70.19)	480(29.81)	230(13.86)	1430(86.14)	40(13.33)	260(86.67)	10(11.11)	80(88.89)	0(0.00)	0(0.00)
Mean	53.80952381	22.85714	25.55556	158.8889	10	65	3.333333	26.66667	0	0
SD	45.76856896	14.54058	22.42271	128.8841	8.164966	12.90994	5.773503	5.773503	0	0

SD: Standard Deviation
